# The Nordic back pain subpopulation program: course patterns established through weekly follow-ups in patients treated for low back pain

**DOI:** 10.1186/1746-1340-18-2

**Published:** 2010-01-15

**Authors:** Alice Kongsted, Charlotte Leboeuf-Yde

**Affiliations:** 1 Nordic Institute of Chiropractic and Clinical Biomechanics, Clinical Locomotion Science, Forskerparken 10A, 5230 Odense M, Denmark; 2 Research Unit for Clinical Biomechanics, University of Southern Denmark, Campusvej 55, 5230 Odense M, Denmark; 3 The Back Research Center, Clinical Locomotion Science, Lindevej 5, 5750 Ringe, Denmark

## Abstract

**Background:**

Low back pain (LBP) is known to have a fluctuating course. In clinical studies, when deciding on duration of treatment and time for follow-up, it is important to know at what point in time a definite pattern of recovery becomes apparent and at what time a possible recurrence is likely to occur. A detailed description of the pain pattern has been difficult to establish with commonly used methods for follow-up, and we now introduce data collection by means of text messaging on mobile phones. The purpose of this study was to describe the detailed course of LBP during 18 weeks in a population treated in the primary care sector by chiropractors.

**Methods:**

The study population consisted of 78 patients presenting to a chiropractor with LBP, who for at least 12 weeks responded to the questions sent by text messaging concerning 1) the number of LBP-days the preceding week and 2) the intensity of present LBP.

**Results:**

A rapid improvement was observed through weeks one to four. After week seven no further improvement happened, and from the 12^th ^week there seemed to be a tendency towards worsening.

**Conclusions:**

We suggest that follow-ups in studies concerning primary sector LBP care are conducted in week seven after treatment was initiated and at some later point which cannot be established from this study. In clinical practice we recommend that patients' LBP status is systematically followed for the first four weeks since lack of improvement during that period should cause watchfulness.

## Background

Low back pain (LBP) is known to have a fluctuating course [1] at least in some groups of patients [2,3]. People with LBP probably seek care when their symptoms are at a peak, and during the ensuing time some will improve, either because of or regardless of the treatment. In clinical studies, when deciding on duration of treatment and time for follow-up, it is important to know at what point in time a definite pattern of recovery becomes apparent. Although this has been shown to happen quite early in the course of treatment [4,5], it is not known at which exact point in time the largest shift occurs. Also, it is not known if this course pattern differs between subgroups of patients, and if so, whether this has any clinical significance.

According to previous cohort studies with 3- and 12-month follow-ups, recurrence of LBP pain appears to be quite common after initial improvement following treatment [3,6-8]. However, as it is not known when to expect pain to recur, it is difficult to determine the optimal points for follow-up assessment in clinical studies. This lack of knowledge also affects clinical practice, as we do not know when patients should be optimally monitored for their long-term outcome. The present methods of data collection (e.g., surveys and clinical registers) are not suitable if we want to identify the cut point for recurrent problems because retrospective data in relation to past fluctuations are likely to have low validity due to memory decay. If instead data were collected frequently at short intervals it would be possible to more accurately capture the turning point and fluctuations of LBP. However, the frequent distribution of questionnaires would be both costly and time-consuming and probably after a while the response rate would become low. Diaries would be a good alternative, providing that they are filled out daily, which is uncertain since they may be filled out in 'lumps' or even when the diary should be returned. Web-based questionnaires would be an excellent alternative but only in people who are computer literate and who frequently open their mail. Fortunately, a new data collection method has been introduced, in which questions are sent to participants as text messages on their mobile phones. Replies are conveyed by means of a new text message. This has made it possible to collect data very frequently on an ongoing basis over a prolonged period of time.

The purpose of this study was to describe the detailed course of LBP during 18 weeks in a population of patients with LBP who were treated in the primary care sector by chiropractors. Specifically, we wanted to answer the following questions: 1) what is the general development of LBP during 18 weeks after treatment has been initiated for a new LBP episode?, 2) at what time is there a major shift towards improvement of symptoms, and at what time - if ever - does this change reverse towards worsening?, and 3) what are the proportions of patients who are recovered each week within a 18 weeks course? Information was presented in two ways: a) in relation to number of days with LBP in the past week, and b) in relation to severity of pain.

## Methods

### Participants

Selected chiropractors in private clinics in one Danish region were invited to participate in the recruitment of patients. Inclusion criteria were: a new episode of LBP with or without sciatica as main complaint (i.e. the patients had not seen the chiropractor for this specific pain episode previous to inclusion), 18 - 65 years, and having a mobile phone. The non-inclusion criteria were: previous back surgery, pregnancy, other significant musculoskeletal problems in addition to the LBP, and inability to read or speak Danish. Prior to inclusion, patients received written and verbal information about the study. The project was presented for the local ethical committee who stated that it did not need approval.

### Clinical procedures

Patients who agreed to participate had a standardised clinical examination. Based on the examination they were classified according to a diagnostic system [9]. According to this system, the possible diagnoses were disc pain, nerve root compression, spinal stenosis, postural syndrome, mechanical dysfunction, sacroiliac joint pain, facet joint pain, abnormal nerve tension, muscle pain and abnormal pain syndrome. Information regarding symptoms duration of the present episode and localisation was collected during the patient history at the first consultation. Further data on aspects of the pain course in relation to mechanical diagnosis and other baseline characteristics will be presented elsewhere. Chiropractors were free to choose whichever treatment they found appropriate.

### LBP registration

Follow-up was conducted by text messages that were automatically sent by a system marketed as "SMS-track"[10]. One SMS (short message service) was sent for each question and the participants replied to the questions by returning a text message. The replies were incorporated into a data file on a server at the SMS-track supplier's office. Follow-up was initiated on the first Sunday following inclusion and thereafter automatically repeated every Sunday for 18 weeks. An automatic reminder was sent if the text message had not been answered on the first coming Thursday. Every week the patients were asked the following questions:

Question 1: 'Please answer how much your lower back hurts today? Choose a number: 0 = no pain at all/1 = some pain/2 = severe pain'. *(Referred to as LBP-intensity)*

Question 2: 'Using a number from 0 to 7, please answer how many days you have been bothered from your lower back this week'. *(Referred to as LBP-days)*

Question 3: 'Using a number from 0 to 7, please answer how many days you have been off work because of your lower back this week. (Answer with X if you are not working)'

### Data analysis

The information from the text messages was automatically incorporated into a spread sheet and afterwards transmitted to STATA 10.1. When answers other than a number were given, data were manually recoded as a number when possible, e.g. "I have no pain" was recoded as 0, and "2 days last week" as 2. Answers that could not be transformed directly into a number were coded as missing values. Data were only included in the analysis from those who had participated at least until the 12^th ^week. Participants were also excluded if they did not participate for three or more weeks in a row during the trial. Since days off work due to LBP (question 3) were infrequent no analysis was made for that item.

Frequency tables were constructed for each of the 18 weeks in relation to LBP-days (answers from 0 to 7). These data were then reduced to three categories: 0 days, 1 - 5 days, and 6 - 7 days, which were transformed into bar graphs and used to visualize the point in time when changes in the number of reported LBP days took place. The reduction of LBP-days into the three categories was done in order to isolate those fully recovered and those with a definite problem, and was supported by the raw plots that indicated that this breakdown would form three distinct groups of patients. The LBP-intensity variable was handled in the same manner, though not reduced into fewer categories.

## Results

### Participants

#### Chiropractors

Seven chiropractors (all women, mean 7.6 years of clinical experience) working in five chiropractic clinics in the northern Danish region included participants for the study. Six of these had graduated from the University of Southern Denmark and one from the Palmer College of Chiropractic, California, USA.

#### Patients

During a four month period from the 19^th ^of February to the 18^th ^of June 2008, 110 patients gave their consent to participate and 101 answered the first text message. From these 69 participated at the 18^th ^and last follow-up. The study population reported on in the present paper consisted of 78 participants (39 men and 39 women, mean age 42.5 years) who participated until at least week 12 with a pause of a maximum of 2 weeks in a row previous to that week. The study population consisted of more females, patients with a shorter duration of pain and more patients without sciatica compared to the group of patients that dropped out. Other parameters did not differ between responders and drop-outs (Table 1). Differences within the study population between those responding all weeks and those missing some answers also appear from Table 1. Those responding every week were more often men and had more frequently consulted the chiropractor with LBP of a short duration than those with some missing answers.

**Table 1:** Comparisons between 1) those who dropped out before week 12, 2) participants who did not answer every week but fulfilled the criteria for being in the study population, and 3) those responding every week.

	1) Dropped out before week 12 n = 32	The study population 2) + 3) n = 78	2) Study population who did not answer all weeks n = 34	3) Study population responding every week n = 44
Gender (%):				
Male	69	50	35	61
Female	31	50	65	39

Age				
(mean [SD])	42.3 [11.5]	42.5 [9.9]	43.0 [9.4]	42.1 [10.3]

Duration of episode (%):				
1 - 7 days	22	45	35	52
8 - 12 weeks	44	33	38	30
> 12 weeks	34	19	21	18
missing	0	3	6	0

Localisation (%):				
LBP	44	55	47	61
LBP + leg pain	53	41	44	39
missing	3	4	9	0

No. of LBP days the 1^st ^week	n = 22	n = 74	n = 30	n = 44
(mean [SD])	4.7 [2.7]	4.9 [2.2]	4.6 [2.4]	5.1 [2.0]

Present pain intensity at the 1^st ^follow-up (%):	n = 24	n = 75	n = 31	n = 44
no pain	29	23	29	18
some	58	61	55	66
severe	13	16	16	16

### Low Back Pain

#### What is the general development of LBP? - A comparison between week one and eighteen

**Figure 1:**
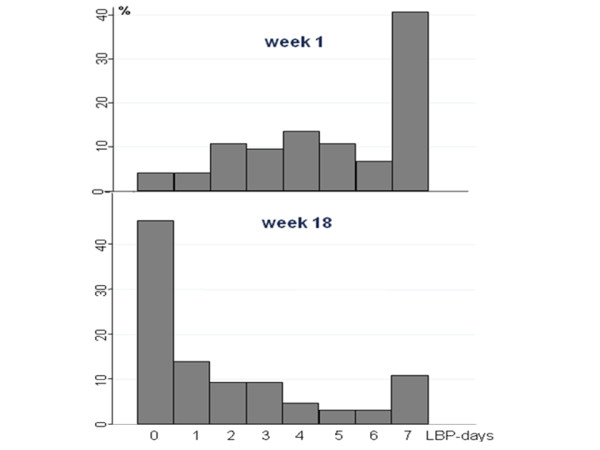
**Number of patients reporting from 0 to 7 days with LBP the preceding week after the 1st (upper graph) and the 18th (lower graph) week**. The upper figure illustrates the distribution of number of pain-days in the first week after consulting a chiropractor whereas the lower figure illustrates the corresponding distribution after 18 weeks.

**Figure 2:**
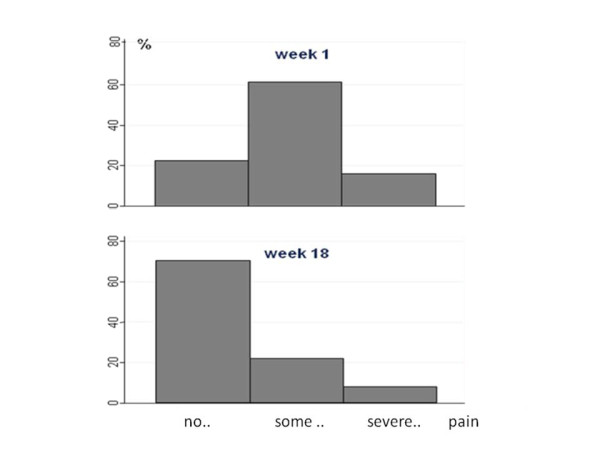
**Number of patients reporting no, some or severe pain on the day of the test message after the 1^st ^(upper graph) and the 18^th ^(lower graph) week**. The upper figure illustrates the distribution of pain intensity in the first week after consulting a chiropractor and the lower figure illustrates the corresponding distribution after 18 weeks.

Fig. 1 shows that the most frequent answer was seven days with LBP the preceding week at the first follow-up, and at the end of follow-up the most frequent response was no days with LBP the preceding week. At the first visit, "some pain" was the LBP-intensity most frequently reported, whereas no pain was the most common response at the end of week 18 (Fig. 2). Three participants reported to have had no days with LBP during the first week.

#### During eighteen weeks, at what time is there a major shift towards improvement of symptoms, and at what time does this reverse towards worsening?

A rapid decline in LBP-days was observed through weeks one to four, and some further reduction in the mean number of LBP-days could be observed until week seven, after which almost no further improvement happened. From the 12^th ^week there was a small tendency towards a higher number of LBP-days again. In the first week participants reported an average of 4.8 days (SD 2.2) with pain, and in the last week 2.0 days (SD 2.4). The mean LBP-intensity followed a similar pattern although the mean values of the pain intensity score should be interpreted with some caution since it covers only three categories.

#### What are the proportions of patients who are recovered, mildly or severely affected throughout the 18 weeks course?

**Figure 3:**
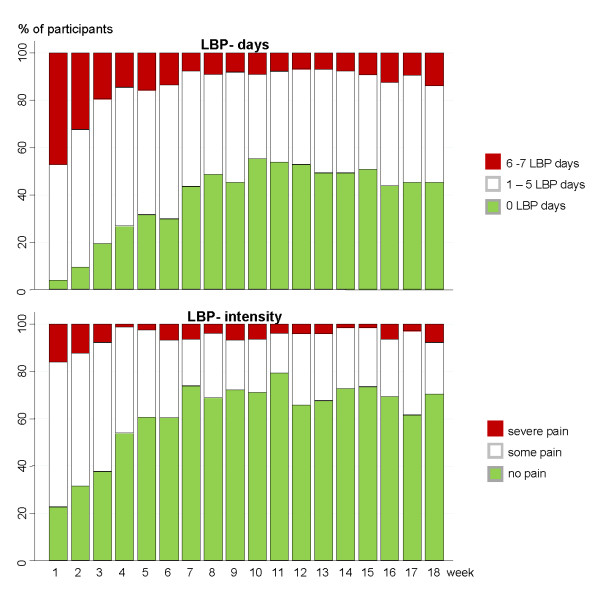
**The percentage of LBP-patients being recovered, mildly and severely affected during a course of 18 weeks**. The graphs illustrate the part of patients reporting no, some and severe symptom during a course of 18 weeks, measured as number of LBP-days (upper) and LBP intensity (lower). Treatment was initiated in the week preceding the first registration.

When grouping participants into those reporting no days of LBP, those with 1 - 5 days and those with 6 - 7 days of LBP, it was observed that the number of participants classified as reporting no days of LBP increased until week 10 and tended to decrease again after the 12^th ^week (Fig. 3). The highest frequencies of pain free participants were in weeks 10 and 12, when 53% reported zero LBP-days. The number of participants with LBP for 6-7 days a week decreased most rapidly until week four, was reduced a little further until the 7^th ^week, and remained relatively stable after that with the lowest frequency from week 12 to week 14. In relation to pain intensity there was a very similar pattern with the proportion of patients reporting no pain increasing until week seven and remaining relatively steady after that; again with a small decline at the end of the period. In the first week, 16% reported severe pain, and that proportion declined during the first 4 weeks, after which it remained small (Fig. 3).

## Discussion

### Results of the study

This appears to be the first study in which weekly follow-ups were performed over a prolonged period of time in patients seeking care for LBP, and thus the first attempt to make a detailed description of the course of LBP following treatment.

We found that the general development of LBP during 18 weeks was improvement both in relation to the number of LBP days in the past week and pain intensity on the day of the follow-up, which resembled each other closely. When interpreting these results it should be noted that "week 1" was not a baseline score, but the reporting of symptoms the first Sunday following the first consultation, i.e. usually after treatment had been initiated. At the beginning, daily LBP was most frequently reported with a gradual shift to no pain days at the end of the 18 weeks. In relation to pain intensity, "some" present pain was by far the most frequent answer in week one, whereas no pain was most frequently reported at the last follow-up.

It was hypothesized that patients seek care when their symptoms are at a peak, and that they therefore will improve in the subsequent period. This hypothesis was supported by the fact that a very quick improvement was observed until week four, which was in line with previous cohort studies on chiropractor patients [4,5]. Whether LBP patients seeking chiropractic care improve rapidly because of or regardless of treatment can of course not be determined with this type of study design.

Our data also indicate that on a group level no further improvement should be expected later than week seven after treatment was initiated. Further, our results tend to support that LBP is a recurrent condition since a slight increase in pain days and pain intensity was observed again after the 12^th ^week. However, a longer follow-up period would be necessary to determine an exact point of time when a possible worsening should be expected to occur.

The highest frequency of being pain free was reached in week ten, when 54% reported no LBP-days, but about half of the patients then keep on experiencing some LBP on and off, and hence do not report complete recovery within a course of 18 weeks.

### Methodological considerations

It was a limitation of the study that we were only able to achieve a 69% response rate at the end of the follow up, but as compared to other primary care studies, we considered this acceptable [2,11,12]. Compared to patients in the secondary sector it may be difficult to motivate primary care patients to spend the time to participate in prospective studies since they, generally, are less troubled by their LBP. Those who dropped out from the study were more frequently men and had a longer duration of symptoms prior to seeing chiropractic care. Age, pain location, LBP-days the first week and LBP-intensity the first week did not differ between the study population completing the study and drop outs. Nonetheless, the longer duration of the current episode in those who dropped out may have affected results although the association between this factor and the prognosis is uncertain [13]. It is possible that a more vigorous information strategy would have helped maintain the interest of the participants throughout the entire study period.

Unfortunately, we did not register how many patients declined to participate or if some potential participants were not invited, and hence we do not know to what extent our results can be generalized to all chiropractic patients. In retrospect, the participating chiropractors estimated that no more than ten patients refused to participate and that only two persons were excluded because they could not use the SMS function. It is also not possible to perform a comparison between the baseline status of this population and other populations treated by chiropractors since we did not collect any pain scores prior to the first treatment. This is a shortcoming in relation to describing the profile of the populations, but did not weaken the answering of our objectives.

The main limitation of the SMS-track method is that only few and simple questions can be presented to the participants at follow-up. In the present study we chose to ask about number of pain days, present pain intensity and number of days sick-listed. We found that sick-listing was not a suitable measure in this population since only few patients had any days with sick-listing. This question could therefore be exchanged for a question on disability, which would provide a more comprehensive picture of the LBP status.

We did not try to fit the curves with any statistical model; instead the course of pain was described by the authors simply from what was visualized in the presented figures. These curves could perhaps be interpreted somehow differently by others, but we have previously shown that it is possible to agree well on visual analyses of individual LBP patterns [14]. Further, statistical methods to identify the shifts in the LBP course were not considered useful since this would be subject to large uncertainties as well with such few observations. Also, this was an initial study intended to be a first step in developing a method for investigating LBP as a fluctuating condition. In that context we find this pragmatic approach relevant, but future full-scale studies should evaluate LBP patterns by established statistical methods for this purpose.

### Recommendations

In relation to follow-up studies concerning primary sector LBP care in which traditional questionnaires are used, we would recommend that the first follow-up takes place in week seven after treatment was initiated to ascertain the short-term level of improvement and around week 12 to observe for early recurrence. Obviously, further knowledge is needed in relation to the need for further follow-up after 12 weeks.

In clinical practice we recommend that patients' LBP status is systematically followed for the first four weeks since fast improvement is expected during that period. Further, the absence of early improvement was previously observed to be associated with a poor long-term outcome [4,12] and clinicians should be aware that no further changes in LBP days or intensity happen later than week seven on a population level. Concerning the timing of secondary prevention, we cannot make any recommendations. Our results indicated that some patients have a recurrence of symptoms around week 12, and it should be explored further whether there is a certain time following a LBP episode when patients are at risk of recurrence and whether any preventive efforts can hinder this.

One should note that recommendations based upon this study apply to a group level. It is necessary to study individual pain patterns in order to identify potentially relevant sub-groups within LBP with different responses to treatment and different pain courses. Such individual patterns within the population reported on here are presented elsewhere [14].

## Conclusions

Weekly follow-ups in a cohort of LBP patients revealed that, on a group level, improvement occurs rapidly after the first consultation to a chiropractor and that no further improvement occurred after the 7^th ^week. Further study is warranted in relation to the long-term development beyond that of week 18.

## Competing interests

The authors declare that they have no competing interests.

## Authors' contributions

Both authors participated in the design of the study, data analysis and drafting of the manuscript. AK instructed the chiropractors who included patients and collected the data. Both authors read and approved the final manuscript.
